# Depressive disorders in older Chinese adults with essential hypertension: A classification tree analysis

**DOI:** 10.3389/fcvm.2022.1035203

**Published:** 2022-10-05

**Authors:** Juan Ruan, Yan-Min Xu, Bao-Liang Zhong

**Affiliations:** ^1^Department of Psychiatry, Wuhan Mental Health Center, Wuhan, China; ^2^Center for Psychological Consultation and Therapy, Wuhan Hospital for Psychotherapy, Wuhan, China

**Keywords:** depressive disorders, hypertension, older adults, classification tree analysis, interaction

## Abstract

**Background:**

Although there has been accumulating evidence on the elevated risk of depression in hypertensive patients, data regarding depressive disorders in older adults with hypertension and the interplay between factors associated with depression in this population are very limited. Disentangling the mutual influences between factors may help illuminate the pathways involved in the pathogenesis of the comorbidity of depression in hypertension. This study investigated the prevalence of depressive disorders in older Chinese adults with hypertension and examined major correlates of depressive disorders and the interactions between correlates by using classification tree analysis (CTA).

**Methods:**

In total, 374 older adults with essential hypertension were enrolled from seven urban and six rural primary care centers in Wuhan, China, and interviewed with the Chinese Mini-international Neuropsychiatric Interview 5.0. Family relationship and feelings of loneliness were assessed with standardized questions. A checklist was used to assess the presence of six major medical conditions: diabetes mellitus, heart disease, cerebrovascular disease, chronic obstructive pulmonary disease, chronic gastric ulcer, and arthritis.

**Results:**

The 1-month prevalence rate of depressive disorders was 25.7%. The CTA model identified four major correlates of depressive disorders: loneliness was the most salient, followed by arthritis, family relationship, and heart disease. There were statistically significant interactions between loneliness and arthritis, loneliness and family relationship, and arthritis and heart disease.

**Conclusion:**

Over one out of every four older Chinese adults with hypertension suffer from depressive disorders. Collaborative multidisciplinary management services are needed to reduce the burden of depression in hypertensive older adults, which may include social work outreach services to promote family relationship, mental health services to relive loneliness, and primary care services to manage arthritis and heart disease.

## Introduction

Although pathophysiological mechanisms underlying the hypertension-depression link are complex and still not fully understood, there has been strong and clear evidence on the elevated risk of depressive symptoms and disorders in hypertensive patients (1–4). For example, in China the prevalence rates of depressive symptoms and DSM-IV depressive disorders among outpatients with essential hypertension in large tertiary general hospitals are 47.6 and 16.6%, which are three and ten times as high as those in the general Chinese population, respectively (5, 6). The co-occurring depression has been associated with prolonged duration of hypertension, poor compliance with antihypertensive agents, and failure of adherence to lifestyle interventions, which in turn, complicates the management of hypertension and increases risk of cardiovascular complications (7–9). In this context, expanding our knowledge on the etiology and mechanisms of depression in hypertensive patients is clinically relevant, which may facilitate the effective management of hypertension and the prevention of hypertension-related complications.

The cause of depression in the general population is multifactorial in nature, which involves biological, psychological, and social factors and their interplays (10–12) and, accordingly, the cause of depression in hypertensive patients is no exception. In the literature, a range of factors associated with depression in hypertensive patients have been reported by many clinical studies in China and many other countries, including female sex, advanced age, a low level of educational attainment, marital status of divorced and widowed, low income, living alone, smoking, alcohol consumption, inadequate social support, a long duration of hypertension, and coexistence of major medical conditions (5, 6, 13–20). Nevertheless, nearly all the available studies focused on the main effects of these factors but none paid attention to how the factors work together to determine the depression risk, which may reveal the mechanisms of the mutual cross-talk between factors and how the combination of factors influences the risk of depression, and, in turn, inform the planning of mental health services. For example, if the influence of living alone on depression is conditional on the sex of an individual: statistically significant association of living alone with depression is evident only in women, providing mental health services to women who were living alone would be more cost-effective. In addition, a further limitation of the prior studies is no findings on the relative contributions of the identified factors to the risk of depression, because the statistical method adopted by previous studies, multiple logistic regression model, is often used to identify statistically significant correlates of depression, not clinically important correlates (21).

Because of the higher prevalence of hypertension in older adults than middle-aged and younger adults, older adults with hypertension are the main target population of interest of many previous studies examining depression in hypertension (13–15, 19, 20). These studies used a variety of self-rating scales of depressive symptoms (i.e., nine-item Patient Health Questionnaire and Zung’s Self-rating Depression Scale) and reported a wide range of prevalence rates of depressive symptoms (12.8–61.0%) in hypertensive older adults (14, 19). However, because of no rigorous psychiatric interviews, the proportion of hypertensive older adults whose depressive symptoms are severe enough to meet the clinical diagnostic criteria of depressive disorders remains unknown (22).

To advance the literature in this area, this study was set out to investigate the prevalence of depressive disorders in older Chinese adults with hypertension, and, adopted classification tree analysis (CTA) to examine the major correlates of depressive disorders and identify the interaction between correlates. Unlike traditional binary logistic regression, CTA is a robust algorithm to identify clinically important factors associated with the outcome of interest and effectively detect factor interactions (23). Furthermore, another strength of CTA is its user-friendly way to show findings on factors associated with the outcome and their interactions, which can be easily applied to routine clinical and primary care practice by healthcare workers with limited statistical understanding (24).

## Materials and methods

### Sample

The study sample was 374 hypertensive older adults from a large-scale multi-center cross-sectional survey that examined mental health and quality of life among a representative sample of older adults receiving primary care in seven urban and six rural primary care centers in Wuhan, China, between October 2015 and November 2016. Older primary care patients who were 65 years or older, voluntary to join the study, and diagnosed with essential hypertension or taking antihypertensive medications were included in the current analysis. Details of the sampling and the recruitment of respondents have been published elsewhere (25–29).

The Ethics Committee of Wuhan Mental Health Center approved the study proposal before the formal survey (approval number WMHC-IRB-S065). All respondents and their guardians (when necessary) provided written informed consent form before the interview.

### Instruments and procedures

The study instrument was a questionnaire, which was administered in a face-to-face format by trained primary care physicians (PCPs). The validated Chinese version of the Mini-international Neuropsychiatric Interview (MINI) 5.0 was used to assess the presence of DSM-IV depressive disorders within the past month, including major depressive disorder, dysthymic disorder, and minor depressive disorder (30).

The demographic variables in the questionnaire were sex, age, education, marital status, self-rated financial status, and residence place. Social factors included living arrangement (alone or not alone), self-rated relationship with family members (good, fair, poor), and self-rated relationship with non-family associates (good, fair, poor). Lifestyle factor was currently smoking, which was defined as smoking 5 days per week or more within the last month (27). Psychological factor was feelings of loneliness, which was assessed with a single-item question: “How often do you feel lonely?” with five answer options: always, often, sometimes, seldom, and never. Participants who felt lonely “sometimes,” “often,” and “always” were those having feelings of loneliness (28). Clinical factors were the comorbid major medical conditions, which was assessed with a checklist and included diabetes mellitus, heart disease, cerebrovascular disease, chronic obstructive pulmonary disease, chronic gastric ulcer, and arthritis.

### Statistical analysis

IBM SPSS statistics software, version 24 (SPSS Inc., Chicago, IL, USA) was used to perform all the analyses. Two-sided *P* < 0.05 was statistically significant. Prevalence rates of depressive disorders and their three subtypes were calculated. By using Chi-square test, we compared the characteristics between respondents with and without depressive disorders to characterize respondents with depressive disorders.

To identify major correlates of depressive disorders and their potential interactions, the exhaustive Chi-squared automatic interaction detection (exhaustive CHAID) growing approach was used to perform the CTA. The target category of the outcome in the CTA was the presence of depressive disorders, and all demographic, social, lifestyle, psychological, and clinical variables were included as input variables. We set the maximum number of layers of growth beneath the root node at three and the minimum node sizes at 50 for parent nodes and 25 for child nodes. The CTA divided the study sample into branch-like segments by comparing Chi-square statistics of all possible categories in relation to depressive disorders and this process continued recursively until the tree was fully grown. These segments consisted of an inverted tree with a root node, internal nodes, and end nodes. Accordingly, the classification tree automatically identified correlates of depressive disorders from the root nodes to endnotes, in the order of importance, as well as the interactions between these correlates (23, 31).

## Results

The average age of the 374 hypertensive older adults was 72.9 years (standard deviation [SD]: 5.8, range: 65–93) and 41.7% were men. Table 1 shows the characteristics of the whole sample and respondents with and without depressive disorders.

**TABLE 1:** Characteristics of hypertensive older Chinese adults, split by the presence and absence of depressive disorders, n (%).

Variable		Total sample (*n* = 374)	Without depressive disorders (*n* = 278)	With depressive disorders (*n* = 96)	χ^2^	*P*
Sex	Male	156 (41.7)	125 (45.0)	31 (32.3)		
	Female	218 (58.3)	153 (55.0)	65 (67.7)	4.713	0.030
Age-groups	65–74 years	235 (62.8)	172 (61.9)	63 (65.6)		
	75 + years	139 (37.2)	106 (38.1)	33 (34.4)	0.431	0.512
Education	Illiterate	79 (21.1)	54 (19.4)	25 (26.0)		
	Primary school	106 (28.3)	70 (25.2)	36 (37.5)		
	Middle school and above	189 (50.5)	154 (55.4)	35 (36.5)	10.365	0.006
Marital status	Married	269 (71.9)	201 (72.3)	68 (70.8)		
	Others*	105 (28.1)	77 (27.7)	28 (29.2)	0.076	0.782
Self-rated economic status	Good	66 (17.6)	56 (20.1)	10 (10.4)		
	Fair	275 (73.5)	207 (74.5)	68 (70.8)		
	Poor	33 (8.8)	15 (5.4)	18 (18.8)	18.376	<0.001
Residence place	Urban	210 (56.1)	168 (60.4)	42 (43.8)		
	Rural	164 (43.9)	110 (39.6)	54 (56.3)	8.065	0.005
Living alone	No	336 (89.8)	250 (89.9)	86 (89.6)		
	Yes	38 (10.2)	28 (10.1)	10 (10.4)	0.009	0.923
Self-rated family relationship	Good	310 (82.9)	241 (86.7)	69 (71.9)		
	Fair and poor**	64 (17.1)	37 (13.3)	27 (28.1)	11.043	0.001
Self-rated non-family relationship	Good	377 (74.1)	212 (76.3)	65 (67.7)		
	Fair and poor**	97 (25.9)	66 (23.7)	31 (32.3)	2.716	0.099
Feelings of loneliness	No	286 (76.5)	226 (81.3)	60 (62.5)		
	Yes	88 (23.5)	52 (18.7)	36 (37.5)	14.009	<0.001
Currently smoking	No	320 (85.6)	234 (84.2)	86 (89.6)		
	Yes	54 (14.4)	44 (15.8)	10 (10.4)	1.691	0.193
Diabetes mellitus	No	287 (76.7)	216 (77.7)	71 (74.0)		
	Yes	87 (23.3)	62 (22.3)	25 (26.0)	0.559	0.455
Heart disease	No	319 (85.3)	246 (88.5)	73 (76.0)		
	Yes	55 (14.7)	32 (11.5)	23 (24.0)	8.815	0.003
Cerebrovascular disease	No	337 (90.1)	257 (92.4)	80 (83.3)		
	Yes	37 (9.9)	21 (7.6)	16 (16.7)	6.647	0.010
Chronic obstructive pulmonary disease	No	351 (93.9)	264 (95.0)	87 (90.6)		
	Yes	23 (6.1)	14 (5.0)	9 (9.4)	2.328	0.127
Chronic gastric ulcer	No	359 (96.0)	273 (98.2)	86 (89.6)		
	Yes	15 (4.0)	5 (1.8)	10 (10.4)	13.767	<0.001
Arthritis	No	343 (91.7)	262 (94.2)	81 (84.4)		
	Yes	31 (8.3)	16 (5.8)	15 (15.6)	9.144	0.002

*“Others” included never married, separated, divorced, widowed, cohabitating, and remarried.

**Because of the very small numbers of the category of “poor” relationship (*n* < 10), “poor” and “fair” were merged into one category.

The 1-month prevalence rate of depressive disorders was 25.7%. The corresponding rates for major depressive disorder, dysthymic disorder, and minor depressive disorder were 13.6, 6.1, and 5.9%, respectively.

As displayed in Table 1, compared to respondents without depressive disorders, depressed respondents were more likely to be women, have an educational attainment of illiterate and primary school, rate their economic status as “poor,” dwell in rural areas, rate their relationship with family members as “fair and poor,” feel lonely, suffer from heart disease, suffer from cerebrovascular disease, suffer from chronic gastric ulcer, and suffer from arthritis (*P* ≤ 0.030).

**FIGURE 1:**
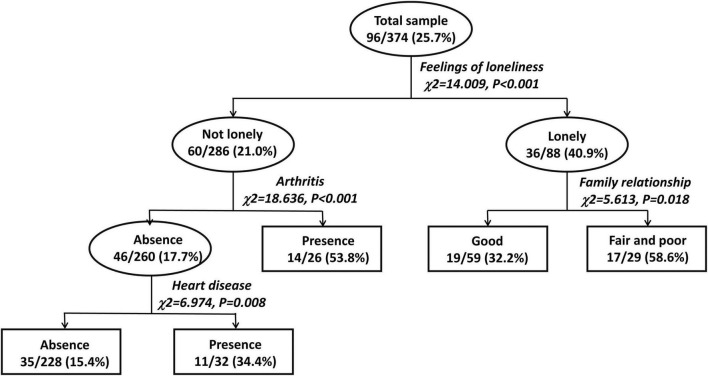
Classification tree analysis for major correlates of depressive disorders and the interactions of factors in older Chinese adults with hypertension.

As shown in Figure 1, the CTA model had three layers of eight nodes, including five end nodes. Four major correlates of depressive disorders were identified: loneliness was the most salient, followed by arthritis, family relationship, and heart disease. Compared to respondents who were not lonely, lonely respondents were 1.9-fold more likely to have depressive disorders (40.9 vs. 21.0%, *P* < 0.001). Among the lonely respondents, relative to those who had good family relationship, those having fair and poor relationship were 1.8-fold more likely to have depressive disorders (58.6 vs. 32.2%, *P* = 0.018). Among respondents who were not lonely, relative to those having no arthritis, those having arthritis were 3.0-fold more likely to have depressive disorders (53.8 vs. 17.7%, *P* < 0.001). Among respondents who were not lonely and did not suffer from arthritis, relative to those having no heart disease, those having heart disease were 2.2-fold more likely to have depressive disorders (34.4 vs. 15.4%, *P* = 0.008). There were statistically significant interactions between loneliness and arthritis, loneliness and family relationship, and arthritis and heart disease.

## Discussion

In China, both hypertension and depressive disorders occur predominantly in the elderly population, and the comorbidity of depression further significantly contributes to the vulnerability of the elderly to hypertension (32). In the context of rapid aging in China, disentangling the complex relationship between factors associated with depression in hypertension may help illuminate the pathways involved in the pathogenesis of the comorbidity of depression, potentially leading the way for effective management of hypertension and effective public health interventions to reduce the disease burden of hypertension (33–37). The present study fills the knowledge gaps by providing empirical data on the prevalence rates of depressive disorders and their subtypes in the elderly population with hypertension and demonstrating major factors associated with depressive disorders and the interactions between these factors. To the best of our knowledge, this is the first study in China examining depressive disorders and testing the interplays between factors associated with depression in older adults with hypertension.

The main findings of this study are the 25.7% prevalence of depressive disorders in hypertensive older adults with major depressive disorder being the most common, four major correlates of depressive disorders with loneliness being the most prominent, and the significant interactions between loneliness and arthritis, loneliness and family relationship, and arthritis and heart disease.

In community-residing older Chinese adults, the 1-month prevalence rates of depressive disorders, major depressive disorder, dysthymic disorder, and mood disorder not otherwise specified (minor depressive disorder is a subtype of this category) were 5.5, 3.8–5.9, 3.9, and 3.0%, respectively (38–40). In older Chinese adults seeking treatment in primary care settings, the 1-month prevalence rates of depressive disorders, major depressive disorder, dysthymic disorder, and minor depressive disorder were 20.3, 10.2–11.3, 4.8, and 5.3%, respectively (26, 41). Therefore, in comparison to these prevalence estimates in older Chinese adults in both community and primary care settings, we found the higher risk of depressive disorders and their subtypes in Chinese patients with hypertension. In the literature, possible explanations for how hypertension results in or exacerbates depression include the mental health burden of suffering from hypertension and its negative impact on a person’s quality of life, a low sense of self-worth, low self-esteem, and a loss of locus of control due to the negative psychological effect of hypertension, and structural changes in brain areas related to emotion as a result of pathophysiologic effects of hypertension on central nervous system (42).

Our findings on possible factors associated with depressive disorders in hypertensive older adults (Table 1) are largely consistent with those from previous studies (5, 6, 13–20). Nevertheless, only four of these factors were finally identified as major correlates of depression in the CTA, suggesting the considerable contributions of loneliness, arthritis, fair and poor family relationship, and heart disease to the elevated risk of depression.

Evidence from longitudinal studies has confirmed the vicious circle between loneliness and depression, that is, loneliness triggers depressive emotions, which create feelings of isolation and alienation and, in turn, result in loneliness (43–45). Accordingly, we replicated the significant loneliness-depression association in hypertensive older adults. In a population-based study of middle-aged adults, Dunlop and colleagues found that both arthritis and heart disease were significantly associated with major depression and the functional limitation caused by the two chronic illnesses can explain their associations with major depression (46). Similar to this study, we also found the significant association of depression with arthritis and heart disease in hypertensive older adults. We also speculate the functional limitation associated with the two major medical conditions might be the primary cause of depressive disorders. Unlike older adults in western countries, family harmony and intergenerational relationship play a pivotal role in the mental well-being of older Chinese adults due to the influence of Confucian culture (47, 48). In accordance with this perspective, fair and poor family relationship was significantly associated with depression in hypertensive older adults.

The three significant interactions between the four major correlates suggest that the factor *per se* not only directly contributes to the risk of depression but also magnifies the negative effects of other factors on the risk of depression. The four factors work together may substantially increase the risk of depression in hypertensive older adults.

This study has several limitations. First, this is a cross-sectional study, so longitudinal studies are warranted to further ascertain the causal relationships between the four identified major correlates and depressive disorders. Second, our CTA is exploratory without evidence of external validity. More studies are needed to validate the findings in other cohorts of older adults with hypertension. Third, the sample size of this study is relatively small. Further, the sample of hypertensive older adults was recruited from primary care settings in Wuhan China. Hypertensive older adults from large general hospitals and other cities in China were not included. Therefore, there might be selection bias in our study sample. Fourth, other factors potentially associated with depression in hypertensive older adults such as personality, physical pain, blood pressure control status, stage of hypertension, and type of antihypertensive drugs were not measured.

In summary, over one out of every four older Chinese adults with hypertension suffer from depressive disorders, suggesting the high risk of depressive disorders in hypertensive older adults. Considering many negative outcomes associated with depression, mental health services for this patient population are urgently needed, which should include psychosocial support, periodic screening for depressive symptoms to ensure early recognition of older adults with depressive disorders, and early initiation of antidepressant treatment when necessary. Our findings on the four major correlates of depressive disorders are clinically interesting because feelings of loneliness, family relationship, arthritis, and heart disease are all potentially modifiable or treatable. The significant interplays between the four major factors further indicate the clinical needs for collaborative multidisciplinary management services for reducing the burden of depression in hypertension, which integrate social work outreach services to promote family relationship, mental health services to relive loneliness, and primary care services to manage arthritis and heart disease.

## Data availability statement

The original contributions presented in this study are included in the article/supplementary material, further inquiries can be directed to the corresponding author.

## Ethics statement

The studies involving human participants were reviewed and approved by the Ethics Committee of Wuhan Mental Health Center. The patients/participants provided their written informed consent to participate in this study.

## Author contributions

JR: acquisition and analysis of data for the study, drafting the manuscript, and interpretation of data for the study. JR and Y-MX: design and acquisition of data for the study. B-LZ: drafting the manuscript, revising the manuscript for important intellectual content, and interpretation of data for the study. All authors contributed to the article and approved the submitted version.
